# Epidemiology in health policies, programs, and services in Brazil: The trajectories and perspectives from ABRASCO's Fifth Strategic Plan for the Development of Epidemiology in Brazil (2025–2029)

**DOI:** 10.1590/1980-549720250005.supl.1

**Published:** 2025-11-17

**Authors:** Laio Magno, Ligia Regina de Oliveira, Maria Rita Donalísio, Gerusa Gibson, Bárbara Campos Silva Valente, Alícia Krüger, Sheila Maria Alvim de Matos, Maria da Glória Teixeira, Alberto Novaes Ramos

**Affiliations:** I Universidade do Estado da Bahia, Department of Life Sciences – Salvador (BA), Brazil.; II Fundação Oswaldo Cruz, Instituto Gonçalo Moniz – Salvador (BA), Brazil.; III Universidade Federal de Mato Grosso, Instituto de Saúde Coletiva, Department of Public Health – Cuiabá (MT), Brazil.; IV Universidade Estadual de Campinas, School of Medical Sciences, Department of Public Health – Campinas (SP), Brazil.; V Universidade Federal do Rio de Janeiro, Instituto de Estudos em Saúde Coletiva – Rio de Janeiro (RJ), Brazil.; VI Fundação Oswaldo Cruz, Escola Politécnica de Saúde Joaquim Venâncio – Rio de Janeiro (RJ), Brazil.; VII Ministry of Health, Secretariat of Health and Environment Surveillance, Office for Inclusion, Diversity, and Equity Policies – Brasília (DF), Brazil.; VIII Universidade Federal da Bahia, Instituto de Saúde Coletiva – Salvador (BA), Brazil.; IX Universidade Federal do Ceará, School of Medicine, Graduate Program in Public Health – Fortaleza (CE), Brazil.

**Keywords:** Epidemiology, Public health, Unified Health System, Health planning, Health evaluation, Health status disparities

## Abstract

**Objective:**

To critically summarize the key topics addressed by the working group "Epidemiology in Health Policies, Programs, and Services" during the development of the Fifth Strategic Plan for the Development of Epidemiology in Brazil (2025–2029), led by the Brazilian Association of Collective Health.

**Methods:**

A historical-contextual analysis was conducted, based on an analytical and comparative review of institutional documents, including previous versions of the Master Plans, scientific literature, and experiences accumulated within the Brazilian Unified Health System (SUS).

**Results:**

The main themes identified were the limitations and potentialities of national health information systems, the challenges related to the production of reliable and high-quality data; the dilemmas involved in training epidemiologists with strong social and political commitments; the need to expand the scope of public health surveillance; the importance of equity-oriented evaluation processes; and the role of intersectoral and territorial articulation in strengthening the field. These discussions were framed within the context of Brazil's demographic, socioeconomic, and political-institutional transformations, highlighting the relevance of critical and ethical epidemiology committed to human rights.

**Conclusion:**

The Master Plans have played a strategic role in articulating science, policy, and practice, thereby consolidating epidemiology as a field of practice, a critical science, and a tool for social transformation. The Fifth Strategic Plan for the Development of Epidemiology in Brazil (2025–2029) emphasizes the necessity of innovative and intersectional approaches to overcoming structural inequalities, inequities, and forms of oppression. This contributes to strengthening the SUS and consolidating an ethical, critical, and socially committed epidemiology.

## INTRODUCTION

Brazilian epidemiology has developed within the dynamic and creative sphere of collective health, extending into related subfields — such as the social and human sciences in health, as well as health policy, planning, and management^1-3^. It has also been closely connected to social movements and the country's redemocratization process through the Brazilian health reform movement, establishing itself as both a theoretical and practical instrument in the construction of the Brazilian Unified Health System (*Sistema Único de Saúde* – SUS)^4,5^.

With the strengthening of health reform and the challenges involved in establishing the Brazilian Association of Collective Health (*Associação Brasileira de Saúde Coletiva* – Abrasco)^1,5^, epidemiology was mobilized to enhance the understanding of population health conditions and to support the development of public policies, particularly in the areas of surveillance and planning^4^. Its scope also broadened beyond disease monitoring, incorporating a more comprehensive concept of health^5-7^.

Over the decades, epidemiology has responded to political and social transformations, including advancements in SUS, democratic setbacks, and the COVID-19 pandemic^1,8-11^. Within this context, the Fifth Strategic Plan for the Development of Epidemiology in Brazil (*V Plano Diretor para o Desenvolvimento da Epidemiologia no Brasil* – V PDDE), prepared by Abrasco for the period 2025–2029 and developed between 2022 and 2024, reaffirms the field's commitment to defending both SUS and democracy, while also redefining the meanings and practices of epidemiology in light of technological transformations, intersectionalities, and current epistemic challenges^12^.

This article sought to critically describe the main themes discussed by the Epidemiology in Health Policies, Programs, and Services Working Group during the development of Abrasco's V PDDE^13^. The analysis builds on the historical foundation of the preceding plans and reflects the transformations experienced in the country, from the redemocratization process to current challenges, including emerging threats to Brazilian democracy.

This study presents a historical-contextual analysis based on an analytical review of institutional documents and related scientific literature, encompassing the trajectory of all versions of the master plans, with particular emphasis on the theme of Epidemiology in Health Policies, Programs, and Services and on the process of developing the V PDDE^13^. The analysis adopted a strategic-situational theoretical-methodological approach, aimed at identifying problems and concrete opportunities for intervention across four interdependent stages: explanatory, normative, strategic, and tactical-operational^14^.

The explanatory phase occurred between 2022 and 2023, during which structured questionnaires were distributed to the community of epidemiologists affiliated with Abrasco's Epidemiology Commission. In August 2023, the commission's working group convened its first in-person meeting in Brasília, Federal District, bringing together public health professionals, researchers, and SUS workers to discuss and systematize the issues identified in the preliminary version of the V PDDE. The normative phase followed in 2024, with the distribution of a new, semi-structured questionnaire to the same community, aimed at developing action proposals to address the identified problems. In September of that year, part of this collective met in Rio de Janeiro, RJ, to deliberate on the proposals and consolidate the normative-propositional version.

The strategic and tactical-operational phases are expected to continue until 2029. The participation of SUS workers was particularly significant in the development of the V PDDE, especially within the working group entitled Epidemiology in Health Policies, Programs, and Services, which was responsible for identifying key challenges and proposing actions for the field of epidemiology.

### Historical foundation

Abrasco has developed five plans, aiming to align the development of epidemiology with the challenges of SUS and the country's social transformations. The evolution of the plan titles — from "Epidemiological Practice in Health Services" (1989) to "Epidemiology in Health Policies, Programs, and Services" (2000 onward) — reflects the expansion of epidemiology's role. Over time, it has progressed from serving primarily as a support tool for health services to becoming a fundamental instrument for policymaking.

#### I PDDE: legacy of the Brazilian health reform movement

The First PDDE (I PDDE – 1989)^15^ emerged in a context of redemocratization, economic crisis, and social mobilization for the right to health. According to Paim^5^, this period was marked by confidence in SUS's potential to promote structural transformations. Within this framework, I PDDE identified epidemiology as a strategic tool for planning and social justice, while also pointing out the underutilization of epidemiological knowledge, weaknesses in information systems, the restriction of surveillance to classic communicable diseases, and the shortage of qualified professionals.

Given this scenario, the document proposed a series of strategies and actions, recognizing that overcoming these challenges would require more than technical solutions. Above all, it called for a political shift: the consolidation of a new epidemiological practice tied to the political decision to effectively implement SUS, capable of integrating knowledge and action to promote a more equitable, effective, and democratic health system. It also proposed strategies for professional training, the development of new indicators, and greater coordination among services, universities, and social movements. Furthermore, it advocated for the integration of scientific and popular knowledge, the democratization of health information, and the redefinition of surveillance to capture the complexity of local conditions and strengthen decentralized management. The document also questioned the excessive reliance on aggregated indicators, which tend to obscure social inequalities, and recommended indicators capable of reflecting distinct social contexts and identifying vulnerable groups. Finally, I PDDE suggested redefining epidemiological surveillance by adopting practices and instruments that capture the complexity of local health conditions, while reinforcing decentralization and the use of epidemiology as a management tool^15^.

#### II PDDE: progress in the implementation of the Unified Health System

The Second PDDE (II PDDE – 1995)^16^ was developed in a context of neoliberal advances and reduced public funding, which posed a threat to the consolidation of SUS^8^. Its formulation coincided with the establishment of the SUS's organic laws, the intensification of municipalization, and the expansion of the Family Health Program. The document emphasized decentralization, public health surveillance, and the creation of the National Center for Epidemiology, which reinforced the use of epidemiology in health actions implemented by states and municipalities.

Despite these advances, several problems persisted, including an insufficient number of qualified professionals, fragmented information systems, limited disease surveillance programs, restricted lists of notifiable conditions, and limited evaluation of the effectiveness of services. For the first time, concern over rising healthcare costs was emphasized, with the use of epidemiological tools proposed as a means to rationalize priorities and optimize resource allocation.

II PDDE also advocated for the incorporation of social determinants and multidisciplinarity. It proposed a set of guidelines ranging from the adoption of broader conceptual definitions to the implementation of practical actions in health services, such as conducting periodic epidemiological surveys and training, and retaining qualified professionals. In addition, it reaffirmed the importance of epidemiology as an essential component of public health management and emphasized that its implementation depended on political decisions, institutional capacity, and structural investments^16^.

#### III PDDE: expansion of the Unified Health System and inequalities

The Third PDDE (III PDDE – 2001)^17^ reinforced the need for a critical, interdisciplinary social epidemiology integrated into the management of SUS. It more broadly incorporated the social determinants of health, drawing on the contributions of the National Commission on Social Determinants of Health, while emphasizing social participation and the democratization of information. The document highlighted advances such as the computerization of information systems, the implementation of local surveys and diagnoses, the surveillance of infant deaths, and the strengthening of oral and environmental health research — supported by the establishment of the Epidemiological and Environmental Surveillance System for SUS. It also underscored the growing use of epidemiological information in health planning and in decision-making processes related to SUS public policies.

However, underfunding, fragmentation of National Health Information Systems (*Sistemas Nacionais de Informação em Saúde* – SNIS) and the fragile institutionalization of health assessment persisted.

The document proposed enhanced coordination between academia and health services, expanded use of geoprocessing techniques for epidemiological data, studies on social inequalities in health, and the institutionalization of professional careers within SUS. It emphasized that consolidating the use of epidemiological knowledge requires not only technical solutions but also enduring political commitments and the integration of public policies at both intra- and intersectoral levels. Thus, it reaffirmed that establishing epidemiology as a strategic instrument for SUS depended on sustained political and institutional support^17^.

#### IV PDDE: hope for change

The Fourth PDDE (IV PDDE – 2005)^18^ emerged amid expectations of social progress and the expansion and capillarization of SUS, despite ongoing crises. It deepened the debate on equity, territory, and social determinants, incorporating environmental and occupational health. The document acknowledged the strengthening of epidemiology within health services and the role of the Public Health Surveillance Secretariat of the Brazilian Ministry of Health, which sought to integrate prevention, surveillance of communicable and noncommunicable diseases, and environmental monitoring. Notable advances included the implementation of rapid response units, the enhancement of SNIS, the creation of the Interagency Health Information Network, and the organization of the National Exhibition of Successful Experiences in Epidemiology, Prevention, and Disease Control. During this period, the offering of professional master's degrees and specialization courses also expanded. IV PDDE further proposed a reorientation of epidemiology grounded in social movements, Southern epistemologies, and popular health practices, reinforcing its commitment to racial, gender, and territorial equity.

The limitations identified in IV PDDE included the limited production of data on vulnerable populations, low capacity to respond to emergencies and chronic diseases, and insufficient coordination between universities and health services. The document also highlighted the fragmentation and weak integration of SNIS, the absence of a common identifier for database linkage, the precarious computing infrastructure within the health service network, and limited capacity for data analysis and utilization. Additional challenges included the scarcity of studies evaluating the effectiveness and impact of interventions, as well as inadequate qualifications and working conditions for health professionals tasked with this work. Finally, the plan acknowledged the limited integration of epidemiological knowledge into intra- and intersectoral policies, which hindered coordinated responses to the social determinants of health^18^.

In this context, the plan recommended the rationalization and integration of SNIS, professional qualification, the strengthening of comprehensive surveillance, the promotion of policy and program evaluation, the recognition and training of health workers, and the intensification of intersectoral coordination and health promotion^18^.

#### V PDDE: resistance and democratic reconstruction

The Fifth PDDE^13^ (V PDDE – 2025) was developed in a context of political and economic crisis, growing social inequalities, and post-COVID-19 pandemic reconstruction. It repositions epidemiology as a tool of resistance and reconstruction, reaffirming its critical and anti-racist character, with a focus on equity, cognitive justice, and the defense of health as a human right. Its historical trajectory reflects the progressive expansion of epidemiology within collective health, consolidating it not only as a technical practice but also as a field of critical practice, capable of responding to diverse circumstances, denouncing inequalities, and proposing political and epistemic alternatives to strengthen SUS.

The following perspectives were discussed in V PDDE from the standpoint of the Epidemiology in Health Policies, Programs, and Services working group.

### National Health Information Systems and epidemiological practices

The expansion of SNIS occurred progressively in response to the needs of SUS, the incorporation of new technologies, and increasing social participation, thereby enhancing the quality, reliability, and representativeness of the data available for management, policy formulation, and scientific research^19,20^.

In this context, V PDDE reaffirms the centrality of health situation analysis, grounded in living conditions, as a critical strategy for addressing health inequalities and ensuring the country's informational sovereignty^13^. Recent initiatives, including the approval of the National Health Information and Informatics Policy^21^ and the establishment of the National Health Data Network (*Rede Nacional de Dados em Saúde* – RNDS)^22,23^, represent significant progress. However, persistent gaps remain, such as underreporting, fragmentation, and technocratic bias^13^. A major challenge is the limited technical and semantic interoperability among multiple SNIS systems, compounded by the absence of unique user identifiers^13^, which hinders longitudinal and integrated analyses^19,24^. Although the RNDS has emerged as a strategic platform, it still lacks effective interoperability with state and municipal systems, as well as integration with other social sectors, including education, social assistance, and the environment.

Furthermore, disparities in infrastructure and personnel across subnational entities limit the qualified use of information, particularly in smaller municipalities, and constrain the scope of comprehensive analyses^13^. Access to data from the private health sector also remains restricted, undermining the completeness of health and epidemiological surveillance^13^. To address these challenges, V PDDE proposes investments to ensure the sustainability of the SNIS and to expand their technical and operational interoperability^13^.

Other critical issues identified include staff training, insufficient epidemiological analyses, and limited capacity for the qualified dissemination of indicators^13^. Consequently, investing in the training of surveillance and management teams, beginning at the undergraduate level, becomes a priority, ensuring both technical mastery of the SNIS and the ability to conduct critical, contextualized analyses in compliance with the General Personal Data Protection Law^25^.

Incomplete data negatively affects policy planning and evaluation. Although new technologies, such as big data and artificial intelligence, offer significant potential, their use requires ethical caution to prevent the exacerbation of inequalities^10,26^ and the marginalization of vulnerable populations^27^. Among the recommended actions, investment in the consolidation and qualification of SNIS platforms within federal entities is emphasized, supporting both management and knowledge production^13^. The plan also advocates for the reaffirmation of national sovereignty over health data, ensuring its public and ethical management^13^.

In this context, health situation analysis must go beyond the mere measurement of morbidity and mortality, incorporating social determinants, territorial inequalities, and markers of social difference, such as race, gender, class, ethnicity, and disability^3,4,12,28,29^. V PDDE also recommends the inclusion of standardized fields for social name, gender identity, race, and disability to ensure equitable analyses that reflect the diversity of the population. Additionally, social participation is emphasized as a fundamental tool for democratizing the management of health information^13^.

The document warns of the risks associated with the privatization of information flows^13^. Consequently, epidemiological analysis must be understood as a political, technical, and ethical practice, committed to transforming health and social realities^30^. By reclaiming this critical legacy, V PDDE proposes that the Brazilian epidemiology community employ information as a tool to combat inequities, thereby strengthening its capacity to support redistributive and emancipatory policies^13^. Addressing these challenges requires continuous investment, technical and scientific training, and expanded social participation in all decision-making bodies within the SUS^31^.

### Training people in epidemiology for the Unified Health System

Epidemiology teaching practices must be aligned with the realities of different territories and the needs of the population. Critical, interdisciplinary, and coordinated training is required, incorporating methodologies that enable contextualized analysis of the health situation^4,26^. The integration of teaching, service, and community emerges as a key strategy to strengthen practical skills, foster innovative responses, and prepare professionals^10^. Accordingly, training should be designed to establish dialogue within local contexts and be grounded in real-world scenarios, integrated with health services and the populations they serve^32^.

In this context, V PDDE highlights a gap between academic training and the actual demands of SUS^13^, which historically oscillates between biomedical and social approaches, with limited emphasis on equity, social determinants, or intersectionality. The document also underscores the absence of structured career paths for public health professionals, weakening the technical and institutional capacity to address health challenges. Additionally, it emphasizes the fragmentation of employment relationships, the precariousness of health work, and the insufficiency of continuing education for SUS employees.

The expansion of innovative and practical training strategies, incorporating new technologies, active methodologies, and tools for translating scientific knowledge into accessible language, is among the solutions proposed by V PDDE^13^. Aligning technical training with public science communication is essential for addressing the infodemic, disinfodemic, and science denialism, phenomena that were exacerbated during the COVID-19 pandemic^10^.

Furthermore, strengthening continuing education, supervision, monitoring, evaluation, training, and communication within SUS contributes to improving management and promoting consistent epidemiological practices, supporting the production of relevant indicators and the analysis of the health situation to inform decision-making at various levels of the system^13^. In addition, training aligned with local realities should be promoted to foster the development of practices committed to improving the living and health conditions of the Brazilian population, particularly in the most vulnerable areas.

### Public health surveillance

Public health surveillance is a strategic component of SUS, aimed at generating knowledge on population health issues, identifying vulnerabilities, controlling risks, responding to health emergencies, and implementing actions capable of reducing health inequalities^31,33^. It also represents a tangible expression of the integration of epidemiology into the daily operations of SUS.

V PDDE emphasizes that integrating surveillance and health care across the various levels of the healthcare network is essential to consolidating a proactive and effective model. This coordination, however, requires institutional redesign and the recognition of surveillance workers, who often operate under precarious employment conditions. The plan also examines historical and structural obstacles within the field while proposing an innovative agenda that repositions surveillance as a democratic and participatory instrument, territorially grounded and guided by the social determinants of health^13^.

Another challenge highlighted in this plan is the limited surveillance of vulnerable populations, including Indigenous peoples, *quilombolas*, individuals experiencing homelessness or deprivation of liberty, LGBTQIAPN+ people, and migrants, among others. The absence of intersectional approaches in surveillance contributes to the epidemiological invisibility of these groups, exacerbating health inequities and neglect^27,34^.

V PDDE proposes the expansion of surveillance modalities, including popular and participatory surveillance, rooted in local territories and guided by community leadership and social control; genomic surveillance, essential for detecting variants of circulating pathogens and emerging microorganisms; and predictive and digital surveillance, emphasizing the ethical use of big data, artificial intelligence, and the monitoring of signals on social media^26^.

Another innovative aspect is the recognition of integrated public health laboratory networks as an essential component of surveillance, requiring investments in infrastructure, supplies, training, and logistics. This measure aligns with the National Health Surveillance Policy and the National Workers’ Health Policy, serving as central guidelines for strengthening surveillance in coordination with primary health care. Additionally, the plan proposes the reconfiguration of surveillance indicators to incorporate multi-criteria and social dimensions capable of guiding equitable actions. This includes the development of social risk matrices, the analysis of territorial vulnerabilities, and the creation of alert systems that consider factors such as racism, environmental and climate conditions, sanitation, and precarious urbanization.

### Assessment

Monitoring and evaluating health programs, services, and interventions are central and strategic components of SUS management, guided by principles of transparency, planning, and effectiveness^35^. This importance is particularly pronounced in contexts characterized by resource scarcity, social inequities, and increasing demands for public accountability.

Despite these advances, V PDDE emphasizes that evaluation has not yet become a routine, participatory, and transformative institutional practice^13^. It further stresses that evaluation should be guided not only by technical considerations but also by democratic values, equity, and engagement with local territories^13^. Among the main obstacles identified are the fragile institutionalization of evaluation and the lack of integration among surveillance, care, and evaluation activities^13^. As a result, evaluation is often reduced to a control and auditing function, undermining its transformative, pedagogical, and political potential^36^.

In this regard, V PDDE emphasizes the need for graduate programs to include research lines focused on the epidemiological evaluation of priority policies and interventions. Such research should extend beyond technical aspects to encompass a critical understanding of public policies, social inequalities, and territorial contexts^35^. Additionally, the plan underscores the importance of incorporating participatory evaluation techniques^13^.

### Insertion of epidemiology into intra- and intersectoral policies

V PDDE proposes a significant shift by calling for epidemiology to operate increasingly beyond disciplinary and sectoral boundaries, contributing to the development of integrated, participatory public policies grounded in the realities of local territories^13^. Accordingly, the incorporation of epidemiology into intra- and intersectoral policies requires transdisciplinarity, co-production of knowledge, and recognition of the epistemological and cultural diversity of the territories. These perspectives are strongly informed by Latin American critical epidemiology, which views health as the outcome of social, historical, and political processes and underscores the need for coordinated intersectoral action to achieve transformation^37,38^.

The document also emphasizes the importance of establishing collaborative networks among undergraduate programs, graduate programs, health services, and civil society as a fundamental strategy for translating health priorities into public policies, grounded in critical outreach and dialogue across knowledge areas. In this framework, the articulation between epidemiology and public policy can occur along three dimensions: analytical (production of intersectoral evidence), institutional (integration across sectors and levels of government), and participatory (engagement of organized civil society and social oversight).

## Conclusions

V PDDE reaffirms epidemiology as a structuring axis of SUS and as a political practice committed to democracy, equity, and social justice. By advocating the strengthening of information systems, the critical and inclusive training of professionals, the reorientation of public health surveillance, and intersectoral integration, the document positions epidemiology as a strategic instrument of resistance and transformation. Beyond serving as a technical guide, it constitutes a political-epistemological project that integrates science, policy, and practice.

## Data Availability

The dataset supporting the results of this study is not publicly available.

## References

[1] Lima N, Santana J (2006). Saúde coletiva como compromisso: a trajetória da Abrasco.

[2] Campos GWS (2000). Saúde pública e saúde coletiva: campo e núcleo de saberes e práticas. Ciênc Saúde Coletiva.

[3] Almeida N (1989). Epidemiologia sem números: uma ciência crítica em ação.

[4] Barata RB (2013). Epidemiologia e políticas públicas. Rev Bras Epidemiol.

[5] Paim JS (2008). A reforma sanitária brasileira e o Sistema Único de Saúde: dialogando com hipóteses concorrentes. Physis.

[6] Donnangelo M (1975). Medicina e sociedade: o médico e seu mercado de trabalho.

[7] Czeresnia D, Freitas CM (2003). Promoção da saúde: conceitos, reflexões, tendências.

[8] Paim J, Travassos C, Almeida C, Bahia L, Macinko J (2011). The Brazilian health system: history, advances, and challenges. Lancet.

[9] Stralen CJ van, Bonfim JR de A (2016). Cebes: democracia é saúde / saúde é democracia. Saúde Debate.

[10] Werneck GL (2023). Epidemiologia e pandemia de COVID-19: oportunidades para rever trajetórias e planejar o futuro. Interface.

[11] Henriques CMP, Moura NFO, Souza PB (2024). Challenges and lessons from the COVID-19 pandemic for Health Surveillance in Brazil: reflections on technologies, models, and system organization. Rev Bras Epidemiol.

[12] Sevalho G (2025). [Social determination of health, complexity, coloniality, and the long term]. Cad Saúde Pública.

[13] Araújo TM (2025). V Plano Diretor para o Desenvolvimento da Epidemiologia no Brasil – 2025–2029. Rev Bras Epidemiol.

[14] Teixeira C (2010). Planejamento em saúde: conceitos, métodos e experiências.

[15] Abrasco (1989). I Plano Diretor para o Desenvolvimento da Epidemiologia no Brasil.

[16] Abrasco (1995). II Plano Diretor para o Desenvolvimento da Epidemiologia no Brasil (1995–1999).

[17] Abrasco (2000). Comissão de Epidemiologia. III Plano diretor para o desenvolvimento da epidemiologia no Brasil 2000-2004. Rev Bras Epidemiol.

[18] Teixeira MG (2005). IV Plano Diretor para o Desenvolvimento da Epidemiologia no Brasil. Rev Bras Epidemiol.

[19] Coelho GC, Chioro A (2021). Afinal, quantos Sistemas de Informação em Saúde de base nacional existem no Brasil?. Cad Saúde Pública.

[20] Coelho GC, Andreazza R, Chioro A (2021). Integração entre os sistemas nacionais de informação em saúde: o caso do e-SUS Atenção Básica. Rev Saúde Pública.

[21] Brasil (2015). Portaria Nº 589.

[22] Moraes JC, França AP, Guibu IA, Barata RB, Grupo ICV (2020). Confiabilidade das informações registradas no Sistema de Informação do Programa Nacional de Imunizações. Epidemiol Serv Saúde.

[23] Brasil (2025). 47-A, caput, § 1º e § 2º, da Lei nº 8.080, de 19 de setembro de 1990.

[24] Costa MVS, Camargos MCS, Viana SMN, Mendes UVS (2025). Avanços e desafios da interoperabilidade no Sistema Único de Saúde. J Health Inform.

[25] Brasil (2018). Lei nº 13.709. Lei Geral de Proteção de Dados Pessoais (LGPD).

[26] Almeida N (2024). Metapresencialidade, saúde digital e saúde coletiva. Interface.

[27] Carneiro S (2011). Racismo, sexismo e desigualdade no Brasil.

[28] Barata RB (2009). Como e por que as desigualdades sociais fazem mal à saúde.

[29] Ayres JRCM (2001). Sujeito, intersubjetividade e práticas de saúde. Ciênc Saúde Coletiva.

[30] Arouca S (2003). O dilema preventivista: contribuição para a compreensão e crítica da medicina preventiva.

[31] Teixeira MG, Costa M, Carmo EH, Oliveira WK, Penna GO (2018). Vigilância em saúde no SUS - construção, efeitos e perspectivas. Ciênc Saúde Coletiva.

[32] Lau B, Duggal P, Ehrhardt S, Armenian H, Branas CC, Colditz GA (2020). Perspectives on the future of epidemiology: a framework for training. Am J Epidemiol.

[33] Monken M, Barcellos C (2005). Vigilância em saúde e território utilizado: possibilidades teóricas e metodológicas. Cad Saúde Pública.

[34] Kalckmann S, Santos CG, Batista LE, Cruz VM (2007). Racismo institucional: um desafio para a eqüidade no SUS?. Saúde Soc.

[35] Hartz Z, Vieira-da-Silva L (2005). Avaliação em saúde: dos modelos teóricos à prática na avaliação de programas e sistemas de saúde.

[36] Cecílio L, Pinheiro R, Mattos RA (2012). As necessidades de saúde como conceito estruturante na luta pela integralidade e equidade na atenção em saúde.

[37] Breilh J (2006). Epidemiologia crítica: ciência emancipadora e interculturalidade.

[38] Laurell A (1982). La salud-enfermedad como proceso social. Cuadernos Médico Sociales.

