# Supplementary material for: Epidemiology in health policies, programs, and services in Brazil: The trajectories and perspectives from ABRASCO's Fifth Strategic Plan for the Development of Epidemiology in Brazil (2025–2029)
Source: Rev Bras Epidemiol. 2025 Nov 17;28(Suppl 1):e250005supl1. doi: 10.1590/1980-549720250005.supl.1 (PMC12622982; doi:10.1590/1980-549720250005.supl.1)
Supplement: Translation

# Translation: Epidemiologia em políticas, programas e serviços de saúde no Brasil: trajetórias e perspectivas do V Plano Diretor para o Desenvolvimento da Epidemiologia no Brasil (2025–2029) da Abrasco

**DOI:** 10.1590/1980-549720250005.supl.1.2

**Authors:** Laio Magno, Ligia Regina de Oliveira, Maria Rita Donalísio, Gerusa Gibson, Bárbara Campos Silva Valente, Alícia Krüger, Sheila Maria Alvim de Matos, Maria da Glória Teixeira, Alberto Novaes Ramos

**Affiliations:** I Universidade do Estado da Bahia, Departamento de Ciências da Vida – Salvador (BA), Brasil.; II Fundação Oswaldo Cruz, Instituto Gonçalo Moniz – Salvador (BA), Brasil.; III Universidade Federal de Mato Grosso, Instituto de Saúde Coletiva, Departamento de Saúde Coletiva – Cuiabá (MT), Brasil.; IV Universidade Estadual de Campinas, Faculdade de Ciências Médicas, Departamento de Saúde Coletiva – Campinas (SP), Brasil.; V Universidade Federal do Rio de Janeiro, Instituto de Estudos em Saúde Coletiva – Rio de Janeiro (RJ), Brasil.; VI Fundação Oswaldo Cruz, Escola Politécnica de Saúde Joaquim Venâncio – Rio de Janeiro (RJ), Brasil.; VII Ministério da Saúde, Secretaria de Vigilância em Saúde e Ambiente, Assessoria de Políticas de Inclusão, Diversidade e Equidade – Brasília (DF), Brasil.; VIII Universidade Federal da Bahia, Instituto de Saúde Coletiva – Salvador (BA), Brasil.; IX Universidade Federal do Ceará, Faculdade de Medicina, Programa de Pós-graduação em Saúde Pública – Fortaleza (CE), Brasil.

**Keywords:** Epidemiologia, Saúde pública, Sistema Único de Saúde, Planejamento em saúde, Avaliação em saúde, Disparidades nos níveis de saúde

## Abstract

**Objetivo:**

Descrever criticamente os principais temas debatidos pelo grupo de trabalho Epidemiologia em Políticas, Programas e Serviços de Saúde durante a construção do V Plano Diretor para o Desenvolvimento da Epidemiologia no Brasil (2025–2029), da Associação Brasileira de Saúde Coletiva.

**Métodos:**

Realizou-se análise histórico-contextual baseada em revisão analítica e comparativa de documentos institucionais, considerando a trajetória das versões anteriores dos planos diretores, literatura científica e experiências acumuladas no Sistema Único de Saúde (SUS).

**Resultados:**

Foram identificados como eixos centrais: as limitações e potencialidades dos sistemas nacionais de informação em saúde; os desafios relacionados à produção de dados e informações qualificadas; os dilemas da formação em epidemiologia comprometida social e politicamente; a necessidade de sentidos ampliados para a vigilância em saúde; a importância de processos avaliativos orientados pela equidade; e a articulação intersetorial e territorial como estratégia de fortalecimento do campo. O debate esteve contextualizado nas transformações demográficas, socioeconômicas e político-institucionais do país, evidenciando a relevância de uma epidemiologia crítica, ética e engajada com os direitos humanos.

**Conclusão:**

Os planos diretores têm desempenhado papel estratégico ao articular ciência, política e prática, consolidando a epidemiologia como campo de prática, ciência crítica e instrumento de transformação social. O V Plano Diretor reforça a necessidade de abordagens inovadoras e interseccionais, comprometidas com a superação das desigualdades, iniquidades e opressões estruturais, contribuindo para o fortalecimento do SUS e para a construção de uma epidemiologia ética, crítica e socialmente implicada.

## INTRODUÇÃO

A epidemiologia brasileira desenvolve-se no espaço pulsante e criativo do campo da saúde coletiva e nas fronteiras com outras subáreas do campo — ciências sociais e humanas em saúde e política, planejamento e gestão em saúde^1-3^. Também esteve articulada a lutas sociais e ao processo de redemocratização do país no movimento da reforma sanitária brasileira, consolidando-se como instrumento teórico e prático de construção do Sistema Único de Saúde (SUS)^4,5^.

Com o fortalecimento da reforma sanitária e os desafios da criação da Associação Brasileira de Saúde Coletiva (Abrasco)^1,5^, a epidemiologia foi sendo mobilizada a contribuir para o conhecimento das condições de saúde da população e subsidiar a formulação de políticas públicas, especialmente nas áreas de vigilância e planejamento^4^. Também foi ampliando seus horizontes para além da vigilância de doenças, incorporando o conceito ampliado de saúde^5-7^.

Ao longo das décadas, a epidemiologia respondeu a transformações políticas e sociais, incluindo avanços do SUS, retrocessos democráticos e a pandemia de COVID-19^1,8-11^. Nesse contexto, o V Plano Diretor para o Desenvolvimento da Epidemiologia no Brasil (PDDE) da Abrasco para o período 2025–2029, construído entre 2022 e 2024, reafirma o compromisso do campo com a defesa do SUS e da democracia, ao mesmo tempo que atualiza sentidos e práticas da epidemiologia à luz de transformações tecnológicas, interseccionalidades e desafios epistêmicos vigentes^12^.

Assim, o presente artigo tem como objetivo descrever criticamente os principais temas debatidos pelo grupo de trabalho Epidemiologia em Políticas, Programas e Serviços de Saúde durante a construção do V PDDE^13^, da Abrasco, com base no acúmulo histórico dos planos que o antecederam, refletindo as transformações do país, desde a redemocratização até os desafios atuais, como as novas ameaças à democracia brasileira.

Trata-se de uma análise histórico-contextual baseada na revisão analítica de documentos institucionais e de literatura científica relacionada, incluindo a trajetória de todas as versões dos planos diretores, com ênfase no tópico Epidemiologia em Políticas, Programas e Serviços de Saúde e no processo de construção do V PDDE^13^. Este adotou abordagem teórico-metodológica estratégico-situacional, voltada para a identificação de problemas e oportunidades reais de intervenção em quatro momentos interdependentes: explicativo, normativo, estratégico e tático-operacional^14^.

O momento explicativo ocorreu entre 2022 e 2023, com envio de questionários estruturados para a comunidade de epidemiologistas vinculados(as) à Comissão de Epidemiologia da Abrasco. Em agosto de 2023, realizou-se o primeiro encontro presencial do grupo de trabalho da comissão, na cidade de Brasília (DF), reunindo sanitaristas, pesquisadores(as) e trabalhadores(as) do SUS para discutir e sistematizar esses problemas na versão preliminar do V PDDE. O momento normativo deu-se em 2024 pelo envio de um novo questionário, dessa vez semiestruturado, à mesma comunidade, visando compor propostas de ações para enfrentar os problemas identificados. Em setembro do mesmo ano, parte desse coletivo se reuniu na cidade do Rio de Janeiro (RJ) para debater proposições e consolidar a versão normativo-propositiva.

Os momentos estratégico e tático-operacional seguirão em curso até 2029. A participação de trabalhadores(as) do SUS se destacou na construção do V PDDE, especialmente no grupo de trabalho intitulado Epidemiologia em Políticas, Programas e Serviços de Saúde, responsável por realizar o reconhecimento dos principais problemas e propostas de ações para a epidemiologia.

### Resgate histórico

A Abrasco elaborou cinco planos, buscando alinhar o desenvolvimento da epidemiologia aos desafios do SUS e às transformações sociais do país. A evolução dos títulos — de "Prática epidemiológica em serviços de saúde" (1989) até "A epidemiologia em políticas, programas e serviços de saúde" (2000 em diante) — expressa a ampliação do seu papel. Ela passou de instrumento de apoio aos serviços de saúde para tornar-se ferramenta fundamental para a formulação de políticas.

#### I PDDE: ecos do movimento de reforma sanitária brasileira

O I PDDE (1989)^15^ surgiu no contexto de redemocratização, crise econômica e mobilização social pelo direito à saúde. Paim^5^ destaca que esse período foi marcado pela crença na capacidade de o SUS induzir transformações estruturais. Nesse contexto, o I PDDE reconhecia a epidemiologia como ferramenta estratégica de planejamento e justiça social, contudo apontava para a subutilização do conhecimento epidemiológico, a fragilidade dos sistemas de informação, a limitação da vigilância às doenças transmissíveis clássicas e a carência de profissionais qualificados.

Diante desse cenário, o documento propôs uma série de estratégias e ações, com reconhecimento de que a superação desses desafios exigiria mais do que soluções técnicas. Reivindicava-se, sobretudo, uma virada política: a consolidação de uma nova prática epidemiológica vinculada à decisão política de implementação efetiva do SUS, capaz de articular conhecimento e ação em prol de um sistema de saúde mais equitativo, resolutivo e democrático. Propunha também estratégias de formação de profissionais, novos indicadores e maior articulação entre serviços, universidades e movimentos sociais. Defendia ainda a integração entre saber científico e popular, a democratização das informações em saúde e a redefinição da vigilância, ampliando seu escopo para captar a complexidade das condições locais e fortalecer a gestão descentralizada. Questionou o uso excessivo de indicadores agregados, que tendem a ocultar desigualdades sociais, defendendo aqueles que refletem as distintas inserções sociais e permitem a identificação de grupos vulneráveis. Por fim, o I PDDE sugeriu a redefinição da vigilância epidemiológica com práticas e instrumentos capazes de captar a complexidade das condições de saúde locais, com descentralização e uso da epidemiologia como instrumento de gestão^15^.

#### II PDDE diretor: avanço da implementação do Sistema Único de Saúde

O II PDDE (1995)^16^ foi elaborado em cenário de avanço neoliberal e redução do financiamento público, que ameaçavam a consolidação do SUS^8^. Sua formulação insere-se no contexto de consolidação das leis orgânicas do SUS, intensificação da municipalização do sistema e expansão do Programa de Saúde da Família. Valorizava a descentralização, a vigilância em saúde e a criação do Centro Nacional de Epidemiologia, que fortalecia o uso da epidemiologia nas ações de saúde desenvolvidas por estados e municípios.

Apesar dos avanços, persistiam problemas como escassez de profissionais qualificados, fragmentação dos sistemas de informação, limitação dos programas de vigilância de doenças e agravos de notificação compulsória e baixa avaliação da efetividade dos serviços. Pela primeira vez, destacou-se a preocupação com os custos crescentes da saúde, propondo o uso de instrumentos epidemiológicos para racionalizar prioridades e recursos.

O II PDDE defendeu, ainda, a incorporação de determinantes sociais e a multidisciplinaridade. Igualmente, propôs um conjunto de diretrizes que vão desde a incorporação de definições conceituais ampliadas até a implementação de ações práticas nos serviços, como a realização de inquéritos epidemiológicos periódicos, a formação e a fixação de profissionais com qualificação. Também reafirmou a importância da epidemiologia como espaço essencial para a gestão pública em saúde e destacou que sua efetivação dependia de decisões políticas, capacidades institucionais e investimentos estruturantes^16^.

#### III PDDE: ampliação do Sistema Único de Saúde e das desigualdades

O III PDDE (2001)^17^ reforçou a necessidade de uma epidemiologia social/crítica, interdisciplinar e articulada à gestão do SUS. Incorporou de forma mais ampla os determinantes sociais da saúde, considerando as contribuições da Comissão Nacional sobre Determinantes Sociais da Saúde e valorizando a participação social e a democratização das informações. Destacou avanços como a informatização dos sistemas, a realização de inquéritos e diagnósticos locais, a vigilância de óbitos infantis, o fortalecimento da pesquisa em saúde bucal e ambiental — com a implementação do Sistema de Vigilância Epidemiológica e Ambiental para o SUS —, bem como o uso cada vez maior da informação epidemiológica no planejamento em saúde e processos decisórios envolvendo políticas públicas do SUS.

Todavia, o subfinanciamento, a fragmentação dos sistemas nacionais de informação em saúde (SNIS) e a frágil institucionalização da avaliação em saúde persistiam.

O documento propôs maior articulação entre academia e serviços, ampliação do uso de técnicas de geoprocessamento de dados epidemiológicos, estudos sobre desigualdades sociais em saúde e institucionalização de carreiras profissionais para o SUS, reafirmando que a consolidação do uso do saber epidemiológico exige também compromissos políticos duradouros, além da garantia de integração das políticas públicas nos âmbitos intra e intersetorial. Reafirmou-se, portanto, que consolidar a epidemiologia como instrumento estratégico para o SUS dependia não apenas de soluções técnicas, mas de um compromisso político-institucional duradouro^17^.

#### IV PDDE: esperança por mudanças

O IV PDDE (2005)^18^ surgiu em meio à expectativa de avanços sociais e de expansão e capilarização do SUS, mesmo em contexto de crises. Aprofundou o debate sobre equidade, território e determinantes sociais, incorporando saúde ambiental e do(a) trabalhador(a). Reconheceu o fortalecimento da epidemiologia nos serviços e do papel da Secretaria de Vigilância em Saúde no Ministério da Saúde, que buscou integrar ações de prevenção, vigilância de doenças transmissíveis e não transmissíveis e vigilância ambiental. Avanços incluíram a implantação de unidades de respostas rápidas, a melhoria dos SNIS, a criação da Rede Interagencial de Informações em Saúde e a realização da Mostra Nacional de Experiências Bem-Sucedidas em Epidemiologia, Prevenção e Controle de Doenças. Ademais, nessa época houve a expansão de oferta de mestrados profissionais e cursos de especialização. O IV PDDE também propôs reorientação da epidemiologia com base nas lutas sociais, nas epistemologias do Sul e nas práticas populares de saúde, reforçando o compromisso da epidemiologia com a equidade racial, de gênero e territorial.

As limitações apontadas no IV PDDE foram a reduzida produção de dados sobre populações vulneráveis, baixa capacidade de resposta a emergências e doenças crônicas, bem como a insuficiente articulação entre universidades e serviços de saúde. Além disso, o documento ressaltou a fragmentação e baixa integração dos SNIS, a inexistência de número identificador comum para relacionamento de banco de dados, a precariedade da infraestrutura computacional da rede de serviços de saúde e a limitada capacidade analítica e de uso dos dados. Outras limitações incluíam a escassez de estudos de avaliação de efetividade e impacto de intervenções, assim como a insuficiente qualificação e condições de trabalho de profissionais de saúde para esse fim. Finalmente, reconheceu-se uma limitada inserção do conhecimento epidemiológico nas políticas intra e intersetoriais, dificultando respostas articuladas aos determinantes sociais da saúde^18^.

Diante desse cenário, recomendaram-se racionalização e integração dos SNIS, qualificação profissional, fortalecimento da vigilância ampliada e estímulo à avaliação de políticas e programas, valorização e qualificação de trabalhadores(as) da saúde e intensificação da articulação intersetorial e da promoção da saúde^18^.

#### V PDDE: resistência e reconstrução democrática

O V PDDE^13^ foi construído em um cenário de crise político-econômica, agravamento das desigualdades sociais e reconstrução pós-pandemia de COVID-19. Ele revisita a epidemiologia como ferramenta de resistência e reconstrução, reafirmando seu caráter crítico, antirracista e voltado à equidade, à justiça cognitiva e à defesa da saúde como direito humano. Ao compor a sua trajetória histórica, reconhece-se a progressiva ampliação da epidemiologia no campo da saúde coletiva, consolidando-a não apenas como prática técnica, mas como campo de práxis crítica, capaz de responder a conjunturas diversas, denunciar desigualdades e propor alternativas políticas e epistêmicas para o fortalecimento do SUS.

A seguir, são abordadas as perspectivas debatidas no V PDDE, sob a lente do grupo de trabalho Epidemiologia em Políticas, Programas e Serviços de Saúde.

### SNIS e práticas epidemiológicas

A expansão dos SNIS ocorreu progressivamente conforme as necessidades do SUS, a incorporação de tecnologia e a crescente participação social, garantindo mais qualidade, confiabilidade e representatividade dos dados disponíveis para a gestão, a formulação de políticas e a produção científica^19,20^.

Nesse sentido, o V PDDE retoma a centralidade da análise de situação de saúde, segundo condições de vida, como estratégia crítica para enfrentamento das desigualdades em saúde e de garantia da soberania informacional do país^13^. Medidas recentes, como a aprovação da Política Nacional de Informação e Informática em Saúde^21^ e a instituição da Rede Nacional de Dados em Saúde (RNDS)^22,23^, representam avanços importantes, entretanto ainda há lacunas persistentes, como subnotificação, fragmentação e viés tecnocrático^13^. Um dos principais entraves é a limitada interoperabilidade técnica e semântica dos múltiplos SNIS, agravada pela ausência de identificadores únicos de usuários(as)^13^, dificultando análises longitudinais e integradas^19,24^. A RNDS, embora emergente como plataforma estratégica, ainda carece de interoperabilidade efetiva com sistemas estaduais e municipais, além de integração com outros setores sociais, como educação, assistência social e meio ambiente.

Além disso, há o destaque para a desigual disponibilidade de infraestrutura e de pessoal nos entes subnacionais limitando o uso qualificado da informação, especialmente em municípios de pequeno porte, restringindo análises abrangentes^13^. Também permanece restrito o acesso a dados do sistema privado, comprometendo a completude da vigilância sanitária e epidemiológica^13^. Para superar essas barreiras, o V PDDE propõe investimentos voltados à sustentabilidade dos SNIS e à ampliação de sua interoperabilidade técnica e operacional^13^.

Outros pontos críticos apontados se referem à formação de pessoal, à insuficiência de análises epidemiológicas e à baixa capacidade de divulgação qualificada de indicadores^13^. Desse modo, torna-se prioritário investir na formação de equipes de vigilância e gestão, desde a graduação, assegurando o domínio técnico dos SNIS e a capacidade de análise crítica e contextualizada, em consonância com a Lei Geral de Proteção de Dados Pessoais^25^.

A incompletude dos dados impacta negativamente no planejamento e na avaliação das políticas. Ainda que novas tecnologias, como megadados e inteligência artificial, tragam potenciais promissores, seu uso exige cautela ética para evitar aprofundamento de desigualdades^10,26^ e invisibilização de populações vulnerabilizadas^27^. Entre as ações recomendadas, destaca-se a necessidade de investimentos para a consolidação e qualificação dos SNIS nos entes federativos para apoio à gestão e à produção de conhecimento^13^. Também é defendida a reafirmação da soberania nacional sobre os dados de saúde com gestão pública e ética^13^.

Nesse contexto, a análise da situação de saúde deve transcender a simples mensuração de morbimortalidade, incorporando determinantes sociais, desigualdades territoriais e marcadores da diferença social, como raça, gênero, classe, etnia e deficiência^3,4,12,28,29^. O V PDDE também recomenda a inserção de campos padronizados para nome social, identidade de gênero, raça e deficiência, a fim de garantir análises equitativas e respeitosas com a diversidade da população. A participação social também é apontada como instrumento fundamental para democratizar a gestão da informação em saúde^13^.

Há uma advertência para os riscos de privatização dos fluxos informacionais^13^. Assim, a análise epidemiológica deve ser compreendida como prática política, técnica e ética, comprometida com a transformação da realidade sanitária e social^30^. Ao retomar essa herança crítica, o V PDDE propõe que a comunidade brasileira de epidemiologistas se aproprie da informação como instrumento de luta contra iniquidades, fortalecendo sua capacidade de subsidiar políticas redistributivas e emancipadoras^13^. Esses desafios demandam investimentos contínuos, qualificação técnico-científica e ampliação da participação social em todas as instâncias decisórias no SUS^31^.

### Formação de pessoas em epidemiologia para o Sistema Único de Saúde

As práticas de ensino da epidemiologia devem estar alinhadas à realidade dos diferentes territórios e demandas da população, sendo necessária uma formação crítica, interdisciplinar e articulada, incorporando metodologias que favoreçam a análise contextualizada da situação de saúde^4,26^. A integração ensino-serviço-comunidade surge como proposta essencial para fortalecer competências práticas, promover respostas inovadoras e formar profissionais^10^. Deve-se, portanto, articular uma formação que estabeleça o diálogo em contextos locais e cuja prática esteja baseada em cenários reais, integrados a serviços de saúde e populações^32^.

Nesse contexto, o V PDDE aponta para uma distância entre a formação acadêmica e as demandas reais do SUS^13^, que oscila historicamente entre enfoques biomédicos e sociais, com limitada ênfase em perspectivas sobre equidade, determinantes sociais ou interseccionalidade. Também destaca a inexistência de carreira estruturada para sanitaristas, fragilizando a capacidade técnica e institucional para enfrentar problemas sanitários. Ressalta adicionalmente a fragmentação de vínculos, a precarização do trabalho em saúde e a insuficiente educação permanente de pessoas no SUS.

A ampliação de estratégias formativas inovadoras e instrumentais, com uso de novas tecnologias, metodologias ativas e ferramentas de tradução do conhecimento científico em linguagem acessível, é uma das soluções propostas pelo V PDDE^13^. O alinhamento da formação técnica à comunicação pública da ciência é um aspecto necessário para enfrentamento de infodemia, desinfodemia e negacionismo científico, fenômenos agravados durante a pandemia de COVID-19^10^.

Ademais, o fortalecimento de educação permanente, a supervisão, o monitoramento, a avaliação, a formação e a comunicação no SUS são ações que contribuem para qualificar a gestão e promover práticas epidemiológicas consistentes, subsidiando a produção de indicadores relevantes e a análise da situação de saúde para apoiar a tomada de decisão nos diferentes níveis do sistema^13^. Adicionalmente, deve-se promover uma formação alinhada às realidades locais que possa contribuir para o desenvolvimento de práticas comprometidas com a transformação das condições de vida e saúde da população brasileira, especialmente nos territórios mais vulnerabilizados.

### Vigilância em saúde

A vigilância em saúde é um dos componentes estratégicos do SUS voltados à produção de conhecimento sobre problemas de saúde da população, ao reconhecimento das vulnerabilidades, ao controle de riscos, à resposta a emergências sanitárias e à promoção de ações capazes de reduzir as desigualdades em saúde^31,33^. Também representa uma das expressões concretas da inserção da epidemiologia no cotidiano do SUS.

O V PDDE destaca que a integração entre vigilância e atenção à saúde nos diferentes pontos da rede de atenção à saúde é fundamental para se consolidar um modelo proativo e resolutivo. Essa articulação, porém, exige redesenho institucional e valorização de trabalhadores(as) da vigilância, que muitas vezes atuam sob vínculos precários. Também aprofunda a análise de entraves históricos e estruturais do campo, ao mesmo tempo que propõe agenda inovadora, que reposiciona a vigilância como instrumento democrático e participativo, com base territorial e orientado pelos determinantes sociais da saúde^13^.

Outro desafio apontado nesse plano é a limitação da vigilância em relação a populações vulnerabilizadas: povos indígenas, quilombolas, pessoas em situação de rua ou privadas de liberdade, LGBTQIAPN+, migrantes, entre outras. A ausência de abordagens interseccionais na vigilância contribui para a invisibilidade epidemiológica dessas populações, aprofundando iniquidades e negligências sanitárias^27,34^.

O V PDDE propõe a ampliação de modalidades de vigilância: vigilância popular e participativa, enraizada nos territórios, com protagonismo comunitário e controle social; vigilância genômica, fundamental para detecção de variantes de patógenos circulantes e microrganismos emergentes; e vigilância preditiva e digital, com uso ético de megadados, inteligência artificial e monitoramento de sinais em mídias sociais^26^.

Outro aspecto inovador é a valorização das redes integradas de laboratórios de saúde pública como parte indissociável da vigilância, o que exige investimentos em infraestrutura, insumos, formação e logística. Essa medida articula-se à defesa da Política Nacional de Vigilância à Saúde e da Política Nacional de Saúde do Trabalhador e da Trabalhadora como diretrizes centrais para fortalecer a vigilância em diálogo com a atenção primária à saúde. Por fim, o plano propõe que os indicadores da vigilância sejam reconfigurados, incorporando dimensões multicritério e sociais capazes de orientar ações equitativas. Isso inclui a construção de matrizes de risco social, análise de vulnerabilidades territoriais e elaboração de sistemas de alerta que considerem, por exemplo, racismo, contexto ambiental e climático, saneamento e urbanização precária.

### Avaliação

O monitoramento e a avaliação de programas, serviços e intervenções em saúde são componentes centrais e estratégicos para a gestão do SUS, pautados em transparência, planejamento e garantia de efetividade^35^. Essa relevância intensifica-se em contextos marcados por escassez de recursos, iniquidades sociais e crescente demanda por responsabilidade pública.

Apesar dos avanços, o V PDDE evidencia que a avaliação ainda não se consolidou como prática institucional rotineira, participativa e transformadora^13^. Além disso, ressalta a necessidade de a avaliação ser orientada não apenas pela técnica, mas também por valores democráticos, equidade e diálogo com territórios^13^. Entre os principais entraves identificados, inserem-se a fragilidade da institucionalização da avaliação e a ausência de integração entre vigilância, atenção e avaliação^13^, reduzindo a avaliação a instrumentos de controle e auditoria, em detrimento de seu potencial transformador, pedagógico e político^36^.

Nesse sentido, o V PDDE indica a necessidade de que programas de pós-graduação incorporem linhas de pesquisa voltadas à avaliação epidemiológica de políticas e intervenções prioritárias. Deve-se, também, ultrapassar o domínio técnico e incluir a compreensão crítica das políticas públicas, das desigualdades sociais e dos contextos territoriais^35^. Adicionalmente, aponta para a necessidade de incorporação de técnicas participativas de avaliação^13^.

### Inserção da epidemiologia em políticas intra e intersetoriais

O V PDDE propõe uma inflexão significativa ao reivindicar que a epidemiologia atue cada vez mais para além das fronteiras disciplinares e setoriais, contribuindo para a produção de políticas públicas integradas, participativas e ancoradas na realidade dos territórios^13^. Dessa maneira, a inserção da epidemiologia em políticas intra e intersetoriais requer transdisciplinaridade, coprodução de conhecimento e valorização da diversidade epistemológica e cultural dos territórios. Essas perspectivas encontram-se fortemente ancoradas em contribuições da epidemiologia crítica latino-americana, que compreende a saúde como resultado de processos sociais, históricos e políticos e que exige uma ação intersetorial coordenada para ser transformada^37,38^.

O documento ressalta ainda a necessidade de construção de redes colaborativas entre cursos de graduação, programas de pós-graduação, serviços e sociedade civil como estratégia fundamental para traduzir prioridades sanitárias em políticas públicas, com base na extensão crítica e no diálogo entre saberes. Assim, a articulação entre epidemiologia e políticas públicas poderia ocorrer em três dimensões: analítica (produção de evidências intersetoriais), institucional (integração entre setores e esferas de governo) e participativa (sociedade civil organizada e controle social).

## Conclusões

O V PDDE reafirma a epidemiologia como eixo estruturante do SUS e prática política comprometida com a democracia, a equidade e a justiça social. Ao propor o fortalecimento dos sistemas de informação, a formação crítica e inclusiva de profissionais, a reorientação da vigilância em saúde e a inserção intersetorial, o documento reposiciona a epidemiologia como instrumento estratégico de resistência e transformação. Mais do que um guia técnico, trata-se de um projeto político-epistemológico que articula ciência, política e prática.

## Data Availability

O conjunto de dados que dá suporte aos resultados deste estudo não está disponível publicamente.
